# A prediction model for predicting the risk of acute respiratory distress syndrome in sepsis patients: a retrospective cohort study

**DOI:** 10.1186/s12890-023-02365-z

**Published:** 2023-03-08

**Authors:** Chi Xu, Lei Zheng, Yicheng Jiang, Li Jin

**Affiliations:** 1 Emergency Department, The Affiliated Wuxi People’s Hospital of Nanjing Medical University, No.299 Qingyang Road, Wuxi, 214023 Jiangsu Province People’s Republic of China; 2 University of California, Santa Cruz, 95064 USA; 3 Emergency Department, The Affiliated Nantong Hospital 3 of Nantong University, Nantong, 226000 People’s Republic of China

**Keywords:** Sepsis, Acute respiratory distress syndrome, MIMIC-IV, Nomogram, Prediction

## Abstract

**Background:**

The risk of death in sepsis patients with acute respiratory distress syndrome (ARDS) was as high as 20–50%. Few studies focused on the risk identification of ARDS among sepsis patients. This study aimed to develop and validate a nomogram to predict the ARDS risk in sepsis patients based on the Medical Information Mart for Intensive Care IV database.

**Methods:**

A total of 16,523 sepsis patients were included and randomly divided into the training and testing sets with a ratio of 7:3 in this retrospective cohort study. The outcomes were defined as the occurrence of ARDS for ICU patients with sepsis. Univariate and multivariate logistic regression analyses were used in the training set to identify the factors that were associated with ARDS risk, which were adopted to establish the nomogram. The receiver operating characteristic and calibration curves were used to assess the predictive performance of nomogram.

**Results:**

Totally 2422 (20.66%) sepsis patients occurred ARDS, with the median follow-up time of 8.47 (5.20, 16.20) days. The results found that body mass index, respiratory rate, urine output, partial pressure of carbon dioxide, blood urea nitrogen, vasopressin, continuous renal replacement therapy, ventilation status, chronic pulmonary disease, malignant cancer, liver disease, septic shock and pancreatitis might be predictors. The area under the curve of developed model were 0.811 (95% CI 0.802–0.820) in the training set and 0.812 (95% CI 0.798–0.826) in the testing set. The calibration curve showed a good concordance between the predicted and observed ARDS among sepsis patients.

**Conclusion:**

We developed a model incorporating thirteen clinical features to predict the ARDS risk in patients with sepsis. The model showed a good predictive ability by internal validation.

**Supplementary Information:**

The online version contains supplementary material available at 10.1186/s12890-023-02365-z.

## Background

Sepsis is defined as a life-threatening organ dysfunction, which caused by a dysregulation of the host’s response to infection [1]. It is estimated that more than 19 million people suffer from sepsis each year, and it has become one of the major threats to human mortality [2]. Acute respiratory distress syndrome (ARDS) is regarded as the earliest and most common complication of sepsis, leading to the excessive and uncontrolled inflammatory reactions and increased mortality rate in sepsis patients, especially for critically ill patients [3, 4]. Previous studies have shown that the risk of death in sepsis patients complicated with ARDS was as high as 20–50% [5, 6]. Therefore, it is essential to pay attention to the risk of ARDS for sepsis patients.

Several researches have indicated that biomarkers, sociodemographic, clinical characteristics were related to the ARDS risk of patients with sepsis [4, 6–8]. In the study of Wang Q et al., they found that microRNA 103 (MIR103) and microRNA 107 (MIR107) were predictive biomarkers for ARDS risks in sepsis patients [6]. A retrospective cohort study found that oral glucocorticoids before admission were associated with a lower incidence of early ARDS among ICU sepsis patients [7]. Nam and colleagues also reported that pneumonia, coagulation score and the central nervous system score were associated with the risk of ARDS in Korean patients with sepsis, and these also were considered as risk factor for 28-day mortality [8]. In general, the risk of developing ARDS in patients with sepsis may be influenced by multiple factors, and the development of predictive models is of great importance for risk assessment [9]. Currently, ARDS risk prediction models for different populations have been proposed [10, 11]. The lung injury prediction score (LIPS) was considered to identify patients at a high risk of ARDS in non-emergency department hospitalized patients [10], as well as patients at high risk for acute lung injury early in the course of their illness and before intensive care unit (ICU) admission [12]. In addition, Lin F, et al. successfully constructed a model combining partial pressure of oxygen: fraction of inspired oxygen (PaO_2_:FiO_2_), platelet count, lactate dehydrogenase, creatinine, and procalcitonin levels to predict the ARDS risk among patients with severe acute pancreatitis [11]. Nevertheless, to the best of our knowledge, there were few studies have established a predictive model by combining multiple predictors to predict the risk of ARDS in sepsis patients.

Herein, the purpose of this study was to develop and validate a prediction model for prediction of ARDS risk in patients with sepsis based on the Medical Information Mart for Intensive Care (MIMIC) IV database.

## Methods

### Source of data

We conducted a retrospective cohort study based on the MIMIC-IV database, as a single-center and freely accessible database, which contains a comprehensive and high-quality data of 53,130 patients in ICU at the Beth Israel Deaconess Medical Center (BIDMC) between 2008 and 2019 [13]. This study used de-identified data and was approved by the Massachusetts Institute of Technology and Institutional Review Board of BIDMC [14]. Patient’s informed consent has been obtained from all participants. All methods were carried out in accordance with relevant guidelines and regulations (declaration of Helsinki).

### Selection of participants

**Fig. 1:**
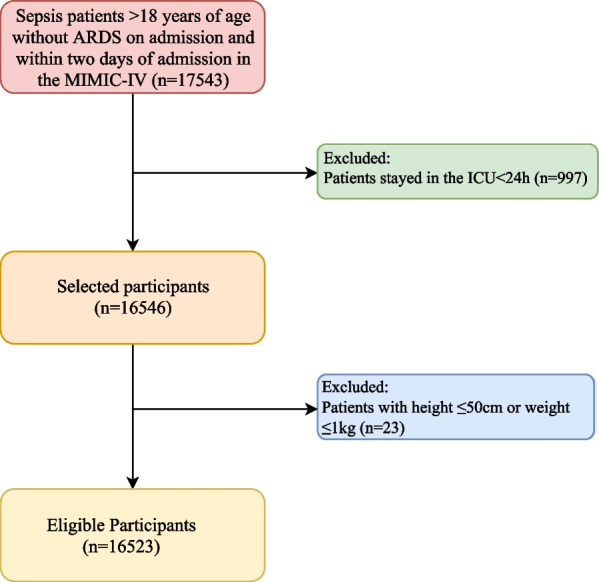
The flow-chart for population selection

Sepsis was defined as a suspected infection combined with an acute increase in SOFA score ≥ 2 according to the Sepsis-3 criteria [1]. All information of participants derived from the MIMIC-IV database. Participants were included in the study if they met the definition of sepsis, were older than 18 years old and did not develop ARDS on admission and within 2 days of admission. The exclusion criteria were as follows: (1) patients who stayed in the ICU less than 24 h; (2) patients who had abnormal data records (height ≤ 50 cm or weight ≤ 1 kg). If patients were admitted repeatedly between 2008 and 2019, we adopted only the record of patient's first admission to the ICU. After implementation of inclusion and exclusion criteria, a total of 16,523 patients with sepsis were included in this study (Fig. 1).

### Data collection

We extracted the following variables from the MIMIC-IV database, including the demographic data [age, gender, ethnicity, marital status, insurance status, admission type, body mass index (BMI, kg/m^2^) and patients’ comorbidity]; the vital signs and laboratory data within 48 h after ICU admission [respiratory rate (times/min), systolic blood pressure (SBP, mmHg), diastolic blood pressure (DBP, mmHg), heart rate (times/min), temperature (℃), urine output (mL), partial pressure of carbon dioxide (PCO_2_, mmHg), FiO_2_, mmHg, bicarbonate (HCO_3_^−^), hemoglobin (g/dL), neutrophil (NEUT), lymphocyte (LYM), platelet (PLT, K/L), white blood cell (WBC, K/L), albumin (ALB), alanine aminotransferase (ALT, U/L), aspartate aminotransferase (AST, U/L), creatinine (mg/dL), blood urea nitrogen (BUN, mg/dL), glucose (mg/dL), C-reactive protein (CRP, mg/L), total cholesterol (TC, mg/dL), triglycerides (TG, mg/dL), low density lipoprotein cholesterol (LDL-C, mg/dL), high density lipoprotein cholesterol (HDL-C, mg/dL)]; severity scoring system [Sequential Organ Failure Assessment (SOFA) score, Simplified Acute Physiology Score (SAPS II)]; medications (heparin, aspirin, antibiotics and vasopressors); treatment [continuous renal replacement therapy (CRRT), mechanical ventilation (MV), red blood cell (RBC) transfusion, PLT transfusion, frozen plasma]. If patients received a laboratory test more than one time during their hospitalization, only the initial test results were included in this study. The diagnosis of ARDS met the Berlin criteria for patients in the MIMIC-IV database [15]. The Berlin criteria include: acute onset, PaO2/FiO2 ≤ 300 mmHg, positive end-expiratory pressure (PEEP) ≥ 5 cm H_2_O on the first day of ICU admission, bilateral infiltrates on chest radiograph, and absence of heart failure [16].

### Outcomes and follow-up

In this retrospective cohort study, the outcomes were defined as the occurrence of ARDS for ICU patients with sepsis. The start date of follow-up was considered as the date of the patient’s admission, and the median follow-up time was 8.47 (5.20, 16.20) days.

### Development and validation of prediction model

All eligible sepsis patients (n = 16,523) were randomly divided into the training (n = 11,566) set and testing set (n = 4957) in a ratio of 7:3. The prediction model was developed in the training set, and validated in the testing set. In the training set, univariate logistic regression analysis was used to screen the factors with *P* < 0.05, combining with factors associated with the risk of ARDS in septic patients in the literature, which were put into a multivariate model for stepwise regression to select some possible predictors. These predictors were used to construct prediction model for predicting the ARDS risk of sepsis patients. The area under the curve (AUC) of receiver operator characteristic curve (ROC) were adopted to compare the predicting performance between constructed prediction model and SOFA, SAPS II scoring system. Calibration curves were used to assess the predicting performance of prediction model in the training set and testing set.

### Statistical analysis

For the present study, mean ± standard deviation (Mean ± SD) and median and quartiles [M (Q1, Q3)] were adopted to described the normally-distributed and nonnormally-distributed of measurement data, respectively. The differences of the groups were compared by the t-test and Mann–Whitney U test. And the categorical data were presented by the number of cases and the constituent ratio [N (%)], and the χ^2^ test performed the comparisons of groups.

We conducted a difference analysis between the training set and testing set. In the training set (n = 11,566), patients with sepsis were divided into ARDS group (n = 2422) and non-ARDS group (n = 9144) according to whether ARDS occurred, and we also did a difference analysis between the ARDS group and non-ARDS group. Lastly, we developed and validated the predicting performance of developed model by ROC and calibration curves. The relative risk (RR) and 95% confidence interval (CI) were calculated. In addition, we deleted the variables with more than 20% missing values (ALT, ALB, TG, LDL-C, HDL-C, AST, NEUT, SaO_2_, TC and CRP), and the multiple filling method was used to fill the variables less than 20% missing values. All analyses were conducted by using SAS 9.4 software (SAS Institute Inc., Cary, NC, USA). *P* < 0.05 was considered to be statistically significant.

## Results

### Baseline characteristics

The incidence of ARDS was 20.66% among total population. No differences were noted between the training set (n = 11,566) and the testing set (n = 4,957) (*P* > 0.05) with respect to baseline information (Additional file 1: Table S1), suggesting that the division of data was balanced and comparable. The characteristics of 11,566 patients with sepsis in the training set were displayed in Table 1, of which 2422 (20.94%) developed ARDS. The sepsis patients developing ARDS had significantly higher heart rate, respiratory rate, BUN level, PCO_2_ level and urine output than sepsis patients with non-ARDS. Additionally, compared to sepsis patients with non-ARDS, those with ARDS were more likely to have chronic pulmonary disease, vasopressin, red blood cell transfusion, liver disease and CRRT (*P* < 0.05).

**Table 1:** Baseline characteristics of 11,566 sepsis patients in the training set

Variables	Total (n = 11,566)	Non-ARDS group (n = 9144)	ARDS group (n = 2422)	Statistics	*P*
Age, years, Mean ± SD	65.45 ± 15.61	66.09 ± 15.53	63.00 ± 15.65	t = 8.69	< 0.001
Gender, n (%)				χ^2^ = 0.115	0.735
Female	4607 (39.83)	3635 (39.75)	972 (40.13)		
Male	6959 (60.17)	5509 (60.25)	1450 (59.87)		
BMI, kg/m^2^, n (%)				χ^2^ = 78.013	< 0.001
< 18.5	334 (2.89)	253 (2.77)	81 (3.34)		
≥ 18.5 and < 25	3322 (28.72)	2733 (29.89)	589 (24.32)		
≥ 25 and < 30	3749 (32.41)	3044 (33.29)	705 (29.11)		
≥ 30	4161 (35.98)	3114 (34.06)	1047 (43.23)		
Ethnicity, n (%)				χ^2^ = 32.109	< 0.001
Black	896 (7.75)	676 (7.39)	220 (9.08)		
Other/unknown	2831 (24.48)	2155 (23.57)	676 (27.91)		
White	7839 (67.78)	6313 (69.04)	1526 (63.01)		
Marital status, n (%)				χ^2^ = 33.155	< 0.001
Married	5448 (47.10)	4418 (48.32)	1030 (42.53)		
Other/unknown	3227 (27.90)	2536 (27.73)	691 (28.53)		
Single	2891 (25.00)	2190 (23.95)	701 (28.94)		
Insurance, n (%)				χ^2^ = 16.907	< 0.001
Medicaid	789 (6.82)	579 (6.33)	210 (8.67)		
Medicare	5238 (45.29)	4149 (45.37)	1089 (44.96)		
Other	5539 (47.89)	4416 (48.29)	1123 (46.37)		
Admission type, n (%)				χ^2^ = 223.075	< 0.001
Elective	739 (6.39)	692 (7.57)	47 (1.94)		
Emergency	5588 (48.31)	4223 (46.18)	1365 (56.36)		
Other	2491 (21.54)	2141 (23.41)	350 (14.45)		
Urgent	2748 (23.76)	2088 (22.83)	660 (27.25)		
SBP, mmHg, M (Q_1_, Q_3_)	118.00 (104.00, 135.00)	118.00 (104.00, 135.00)	119.00 (103.00, 137.00)	Z = 0.658	0.511
DBP, mmHg, M (Q_1_, Q_3_)	64.00 (54.00, 76.00)	63.00 (54.00, 75.00)	66.00 (55.00, 79.00)	Z = 5.887	< 0.001
Temperature, ℃, Mean ± SD	36.63 ± 0.95	36.60 ± 0.92	36.74 ± 1.06	t = − 6.13	< 0.001
Heart rate, times/min, Mean ± SD	89.37 ± 20.02	87.83 ± 19.18	95.20 ± 21.98	t = − 15.07	< 0.001
Respiratory rate, times/min, M (Q_1_, Q_3_)	18.00 (15.00, 22.00)	17.00 (14.00, 22.00)	21.00 (17.00, 25.00)	Z = 23.702	< 0.001
Urine output, mL, M (Q_1_, Q_3_)	2990.00 (1885.00, 4400.00)	2810.00 (1835.00, 4020.00)	4110.00 (2335.00, 5590.00)	Z = 21.158	< 0.001
PCO_2_, mmHg, Mean ± SD	41.00 (36.00, 46.00)	40.00 (36.00, 46.00)	42.00 (35.00, 50.00)	Z = 6.887	< 0.001
FiO_2_, mmHg, M (Q_1_, Q_3_)	100.00 (50.00, 100.00)	100.00 (50.00, 100.00)	80.00 (50.00, 100.00)	Z = − 2.278	0.023
HCO_3−_, Mean ± SD	22.52 ± 4.67	22.64 ± 4.37	22.09 ± 5.64	t = 4.41	< 0.001
Hemoglobin, g/dL, Mean ± SD	11.38 ± 2.28	11.46 ± 2.24	11.09 ± 2.40	t = 6.81	< 0.001
PLT, K/L, M (Q1, Q3)	189.00 (135.00, 252.00)	188.00 (137.00, 250.00)	191.00 (129.00, 259.00)	Z = − 0.126	0.900
WBC, K/L, M (Q1, Q3)	10.60 (7.40, 15.10)	10.40 (7.30, 14.70)	11.50 (7.80, 16.80)	Z = 7.740	< 0.001
Creatinine, mg/dL, M (Q1, Q3)	1.00 (0.80, 1.50)	1.00 (0.80, 1.40)	1.10 (0.80, 1.80)	Z = 9.149	< 0.001
BUN, mg/dL, M (Q_1_, Q_3_)	20.00 (14.00, 32.00)	19.00 (14.00, 30.00)	23.00 (15.00, 39.00)	Z = 9.392	< 0.001
Glucose, mg/dL, M (Q_1_, Q_3_)	123.00 (101.00, 162.00)	120.00 (100.00, 158.00)	132.00 (105.00, 176.00)	Z = 9.068	< 0.001
SOFA, M (Q_1_, Q_3_)	39.00 (31.00,49.00)	37.00 (30.00,47.00)	44.00 (35.00,55.00)	Z = 19.036	< 0.001
SAPS II, M (Q_1_, Q_3_)	2.00 (0.00,4.00)	2.00 (0.00,4.00)	2.00 (0.00,4.00)	Z = 3.294	< 0.001
Vasopressin, n (%)				χ^2^ = 687.639	< 0.001
No	9989 (86.37)	8291 (90.67)	1698 (70.11)		
Yes	1577 (13.63)	853 (9.33)	724 (29.89)		
CRRT, n (%)				χ^2^ = 638.980	< 0.001
No	10,682 (92.36)	8739 (95.57)	1943 (80.22)		
Yes	884 (7.64)	405 (4.43)	479 (19.78)		
Ventilation status, n (%)				χ^2^ = 675.707	< 0.001
High flow	557 (4.82)	532 (5.82)	25 (1.03)		
Invasive vent	114 (0.99)	73 (0.80)	41 (1.69)		
Non-invasive vent	2115 (18.29)	1258 (13.76)	857 (35.38)		
Oxygen	73 (0.63)	61 (0.67)	12 (0.50)		
Trach	8707 (75.28)	7220 (78.96)	1487 (61.40)		
RBC-transfusion, n (%)				χ^2^ = 229.181	< 0.001
No	6855 (59.27)	5745 (62.83)	1110 (45.83)		
Yes	4711 (40.73)	3399 (37.17)	1312 (54.17)		
PLT-transfusion, n (%)				χ^2^ = 91.898	< 0.001
No	9860 (85.25)	7944 (86.88)	1916 (79.11)		
Yes	1706 (14.75)	1200 (13.12)	506 (20.89)		
Frozen plasma, n (%)				χ^2^ = 235.718	< .001
No	10,069 (87.06)	8186 (89.52)	1883 (77.75)		
Yes	1497 (12.94)	958 (10.48)	539 (22.25)		
Diabetes, n (%)				χ^2^ = 0.543	0.461
No	7965 (68.87)	6312 (69.03)	1653 (68.25)		
Yes	3601 (31.13)	2832 (30.97)	769 (31.75)		
Chronic pulmonary disease, n (%)				χ^2^ = 57.827	< 0.001
No	8464 (73.18)	6839 (74.79)	1625 (67.09)		
Yes	3102 (26.82)	2305 (25.21)	797 (32.91)		
Renal disease, n (%)				χ^2^ = 2.407	0.121
No	9115 (78.81)	7234 (79.11)	1881 (77.66)		
Yes	2451 (21.19)	1910 (20.89)	541 (22.34)		
Malignant cancer, n (%)				χ^2^ = 11.189	< 0.001
No	10,179 (88.01)	8095 (88.53)	2084 (86.04)		
Yes	1387 (11.99)	1049 (11.47)	338 (13.96)		
Liver disease, n (%)				χ^2^ = 179.186	< 0.001
No	9829 (84.98)	7980 (87.27)	1849 (76.34)		
Yes	1737 (15.02)	1164 (12.73)	573 (23.66)		
Myocardial infarct, n (%)				χ^2^ = 0.349	0.555
No	9282 (80.25)	7328 (80.14)	1954 (80.68)		
Yes	2284 (19.75)	1816 (19.86)	468 (19.32)		
Leukemia, n (%)				χ^2^ = 3.859	0.049
No	11,379 (98.38)	9007 (98.50)	2372 (97.94)		
Yes	187 (1.62)	137 (1.50)	50 (2.06)		
Septic shock, n (%)				χ^2^ = 109.800	< 0.001
No	10,922 (94.43)	8740 (95.58)	2182 (90.09)		
Yes	644 (5.57)	404 (4.42)	240 (9.91)		
Pancreatitis, n (%)				χ^2^ = 120.732	< 0.001
No	11,311 (97.80)	9013 (98.57)	2298 (94.88)		
Yes	255 (2.20)	131 (1.43)	124 (5.12)		

*BMI* Body mass index; *SBP* Systolic blood pressure; *DBP* Diastolic blood pressure; *SPO*_*2*_ Pulse oxygen saturation; *PCO*_*2*_ Partial pressure of carbon dioxide; *PO*_*2*_ Oxygen partial pressure; *FiO*_*2*_ Fraction of inspired oxygen; *HCO*_*3*_^*−*^ Bicarbonate; *PLT* Platelet; *WBC* White blood cell; *BUN* Blood urea nitrogen; *SOFA* Sequential organ failure assessment; *SAPS II* Simplified acute physiology score II; *CRRT* Continuous renal replacement therapy; *RBC* Red blood cell; *PLT* Platelets; *ARDS* Acute respiratory distress syndrome

### Construction of the prediction model

**Fig. 2:**
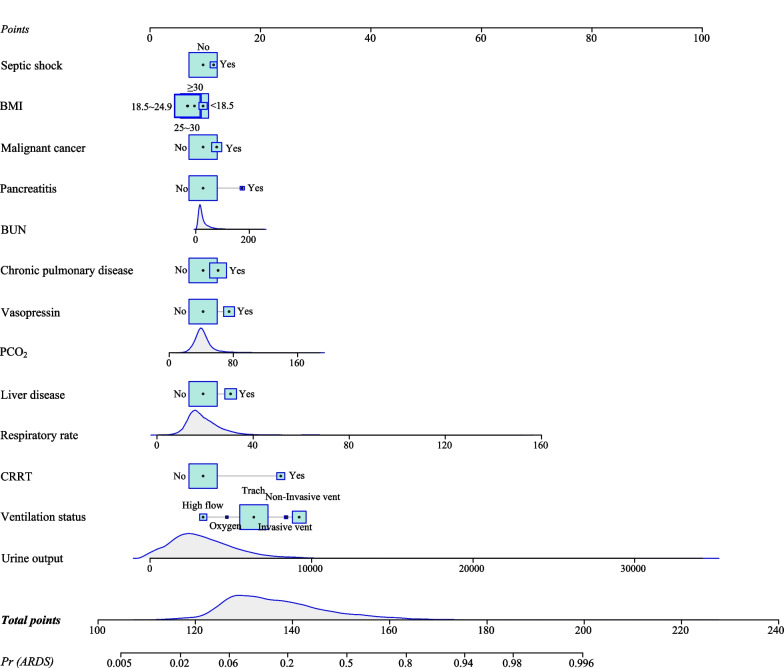
The nomogram for predicting the ARDS risk in ICU patients with sepsis

**Fig. 3:**
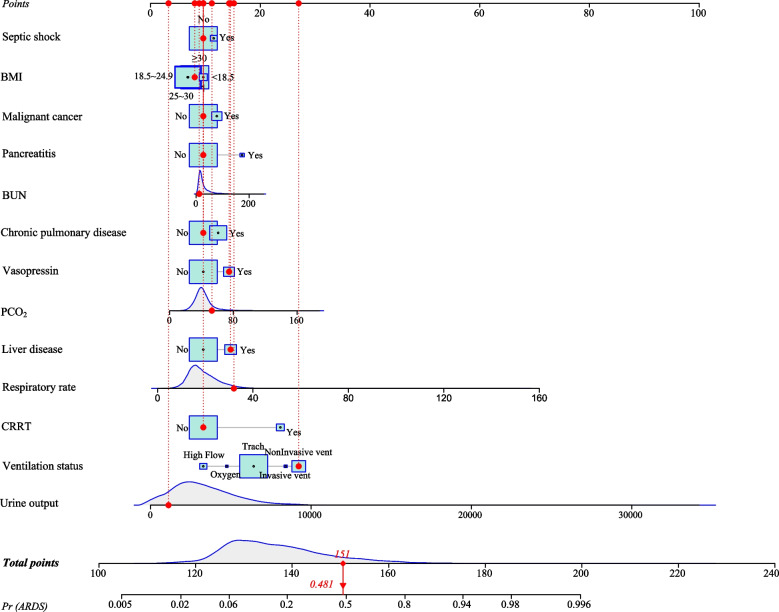
An example for the application of the nomogram

The multivariate logistic regression analysis in the training set found that BMI, respiratory rate, urine output, PCO_2_, BUN, vasopressin, CRRT, ventilation status, chronic pulmonary disease, malignant cancer, liver disease, septic shock and pancreatitis might be predictors (Table 2). A prognostic prediction model, containing thirteen prognostic factors, to predict the ARDS risk in sepsis patients was established. For visualizing the prediction model, we plotted a nomogram (Fig. 2). For instance, a patient with sepsis had a septic shock (No), malignant cancer (No), BMI ≥ 30 kg/m^2^, BUN = 12 mg/dL, pancreatitis (No), chronic pulmonary disease (No), vasopressin (Yes), liver disease (Yes), PCO_2_ = 53 mmHg, respiratory rate = 32 times/min, CRRT (No), ventilation status = non-invasive vent, urine output = 1120 mL, the total score was 151 points and meant a predicted the risk of ARDS of 0.481, which was consistent with the actual outcome of this patient with sepsis (Fig. 3). Additionally, we also have developed an online prediction nomogram for easy clinical application: https://xuchi777.shinyapps.io/DynNomapp/

**Table 2:** The prognostic factors associated with the risk of ARDS for patients with sepsis

Variables	Univariate logistic regression model		Multivariate logistic regression model	
	RR (95% CI)	*P*	RR (95% CI)	*P*
Age	0.988 (0.985–0.990)	< 0.001	–	–
Gender				
Female	Ref		–	–
Male	0.984 (0.898–1.079)	0.734	–	–
BMI				
< 18.5	Ref		Ref	
≥ 18.5 and < 25	0.673 (0.516–0.878)	0.003	0.732 (0.542–0.987)	0.041
≥ 25 and < 30	0.723 (0.556–0.941)	0.016	0.748 (0.555–1.008)	0.057
≥ 30	1.050 (0.810–1.362)	0.712	0.863 (0.642–1.159)	0.326
Ethnicity				
White	Ref		–	–
Black	1.346 (1.145–1.583)	< 0.001	–	–
Other/unknown	1.298 (1.171–1.438)	< 0.001	–	–
Marital status				
Single	Ref		–	–
Married	0.728 (0.653–0.812)	< 0.001	–	–
Other/unknown	0.851 (0.755–0.959)	0.008	–	–
SBP	0.988 (0.927–1.053)	0.715	–	–
DBP	0.990 (0.933–1.051)	0.748	–	–
Respiratory rate	1.075 (1.067–1.082)	< 0.001	1.049 (1.041–1.058)	< 0.001
Urine output	1.635 (1.563–1.710)	< 0.001	1.000 (1.000–1.000)	< 0.001
PCO_2_	1.019 (1.015–1.022)	< 0.001	1.017 (1.013–1.021)	< 0.001
FiO_2_	0.998 (0.996–1.000)	0.016	–	–
HCO_3_^−^	0.975 (0.966–0.985)	< 0.001	–	–
Hemoglobin	0.931 (0.913–0.950)	< 0.001	–	–
PLT	1.017 (0.973–1.063)	0.459	–	–
WBC	1.015 (1.010–1.019)	< 0.001	–	–
Creatinine	1.076 (1.048–1.104)	< 0.001	–	–
BUN	1.008 (1.006–1.010)	< 0.001	1.005 (1.002–1.007)	< 0.001
Glucose	1.002 (1.001–1.002)	< 0.001	–	–
Vasopressin				
No	Ref		Ref	
Yes	4.144 (3.705–4.635)	< 0.001	1.711 (1.491–1.964)	< 0.001
CRRT				
No	Ref		Ref	
Yes	5.319 (4.619–6.126)	< 0.001	4.870 (4.054–5.851)	< 0.001
Ventilation status				
High flow	Ref		Ref	
Invasive vent	11.951 (6.866–20.801)	< 0.001	5.617 (3.122–10.107)	< 0.001
Non-invasive vent	14.495 (9.616–21.850)	< 0.001	7.387 (4.825–11.308)	< 0.001
Oxygen	4.186 (2.002–8.752)	< 0.001	1.681 (0.751–3.760)	0.206
Trach	4.382 (2.923–6.570)	< 0.001	2.906 (1.914–4.412)	< 0.001
PLT-transfusion				
No	Ref		–	–
Yes	1.748 (1.558–1.962)	< 0.001	–	–
Diabetes				
No	Ref		–	–
Yes	1.037 (0.942–1.142)	0.460	–	–
Chronic pulmonary disease				
No	Ref		Ref	
Yes	1.455 (1.321–1.604)	< 0.001	1.353 (1.208–1.514)	< 0.001
Renal disease				
No	Ref		–	–
Yes	1.089 (0.978–1.214)	0.121	–	–
Malignant cancer				
No	Ref		Ref	
Yes	1.252 (1.097–1.428)	< 0.001	1.278 (1.097–1.488)	0.002
Liver disease				
No	Ref		Ref	
Yes	2.125 (1.899–2.377)	< 0.001	1.720 (1.505–1.967)	< 0.001
Leukemia				
No	Ref		–	–
Yes	1.386 (1.000–1.922)	0.050	–	–
Myocardial infarct				
No	Ref		–	–
Yes	0.967 (0.863–1.083)	0.557	–	–
Septic shock				
No	Ref		Ref	
Yes	2.380 (2.015–2.811)	< 0.001	1.268 (1.044–1.541)	0.017
Pancreatitis				
No	Ref		Ref	
Yes	3.713 (2.892–4.766)	< 0.001	2.273 (1.693–3.053)	< 0.001

*BMI* Body mass index; *SBP* Systolic blood pressure; *DBP* Diastolic blood pressure; *SPO*_*2*_ Pulse oxygen saturation; *PCO*_*2*_ Partial pressure of carbon dioxide; *PO*_*2*_ Oxygen partial pressure; *FiO*_*2*_ Fraction of inspired oxygen; *HCO*_*3*_^*−*^ Bicarbonate; *PLT* Platelet; *WBC* White blood cell; *BUN* Blood urea nitrogen; *CRRT* Continuous renal replacement therapy; *RBC* Red blood cell; *PLT* Platelets; *ARDS* Acute respiratory distress syndrome; *RR* Relative risk; *CI* confidence interval

### Validation of the prediction model

**Fig. 4:**
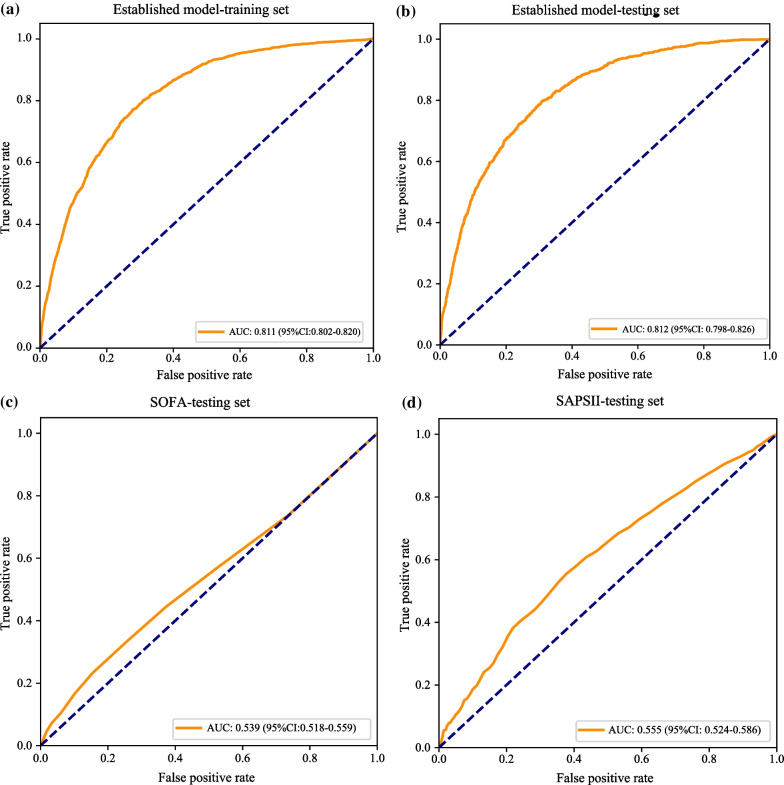
ROC curves of **a** established model in the training set; **b** established model in the testing set; **c** SOFA in the testing set; **d** SAPSII in the testing set

**Fig. 5:**
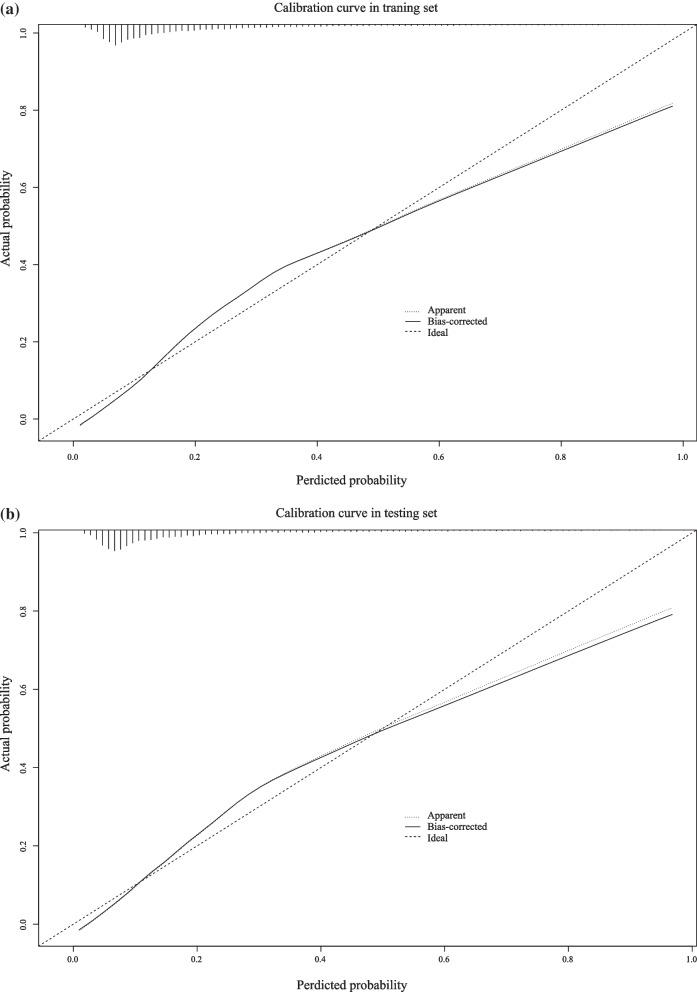
Calibration curves of **a** the training set and **b** testing set

To assess the predictive ability of developed prediction model, the ROC curves and calibration curves were applied in this study. As presented in Table 3, the accuracy, sensitivity, specificity, PPV and NPV of prediction model was 0.732 (95% CI 0.724–0.740), 0.762 (95% CI 0.745–0.779), 0.724 (95% CI 0.714–0.733), 0.422 (95% CI 0.407–0.437) and 0.920 (95% CI 0.914–0.926) respectively, in the training set. Similarly, Table 3 displays that the established model had a 0.705 (95% CI 0.692–0.718) of accuracy, 0.798 (95% CI 0.773–0.823) of sensitivity, 0.682 (95% CI 0.668–0.697) of specificity, 0.385 (95% CI 0.364–0.407) of PPV and 0.931 (95% CI 0.922–0.940) of NPV in the testing set. Moreover, Table 3 also showed that the area under the curve (AUC) of the constructed prediction model was 0.811 (95% CI 0.802–0.820) in the training set (Fig. 4a), corresponding to 0.812 (95% CI 0.798–0.826) in the testing set (Fig. 4b). We also compared the predicting value of constructed prediction model and SOFA, SAPS II scoring systems for predicting the ARDS risk for sepsis patients (Table 3). The AUC of SOFA score and SAPS II score was 0.539 (95% CI 0.518–0.559) and 0.609 (95% CI 0.589–0.629) in the testing set (Fig. 4c and d), separately, which was obviously lower than constructed prediction model (*P* < 0.001). The result implied that the constructed prediction model had favorable discriminatory ability for the prediction of ARDS risk in patients with sepsis. In addition, the calibration curve also showed a good concordance between the predicted and observed risk of ARDS in both training and testing sets (Fig. 5a and b).

**Table 3:** The predictive performance of prediction model, SOFA and SAPSII

Models	Sets	Accuracy (95% CI)	AUC (95% CI)	Sensitivity (95% CI)	Specificity (95% CI)	PPV (95% CI)	NPV (95% CI)
Established model	Testing set	0.705 (0.692–0.718)	0.812 (0.798–0.826)	0.798 (0.773–0.823)	0.682 (0.668–0.697)	0.385 (0.364–0.407)	0.931 (0.922–0.940)
	Training set	0.732 (0.724–0.740)***	0.811 (0.802–0.820)	0.762 (0.745–0.779)*	0.724 (0.714–0.733)***	0.422 (0.407–0.437)**	0.920 (0.914–0.926) *
SOFA	Testing set	0.723 (0.711–0.736)	0.539 (0.518–0.559)***	0.232 (0.206–0.258)***	0.846 (0.835–0.857)***	0.273 (0.243–0.304)***	0.815 (0.803–0.827)***
SAPSII	Testing set	0.609 (0.595–0.623)***	0.609 (0.589–0.629)***	0.555 (0.524–0.586)***	0.623 (0.607–0.638)***	0.269 (0.249–0.288)***	0.848 (0.835–0.861)***

*AUC* The area under of curve; *CI* Confidence interval; *SOFA* Sequential organ failure assessment; *SAPS II* Simplified acute physiology score II; *PPV* Positive predictive value; *NPV* Negative predictive value

Taking established model-testing set as reference, the predictive performance of established model training set, SOFA-testing set and SAPSII-testing set was compared;

*represents *P* < 0.05; **represents *P* < 0.01; ***represents *P* < 0.001

## Discussion

In this retrospective cohort study, a prediction model for predicting the ARDS risk in sepsis patients admitted to ICU was developed. Through verification, this model had a good predictive ability as well as discrimination.

ARDS is considered to be a serious and acute inflammatory lung injury, and could increase the severity of illness and brought a worse outcome for patients with sepsis [17]. Zhao J, et al. pointed out that sepsis-associated ARDS has a higher disease severity and worse clinical outcomes than non-sepsis-associated ARDS [18]. Therefore, early identification of patients with sepsis who are at high risk of developing ARDS is very important. Previous research has found that some prediction model for predicting the ARDS risk were developed and validated in traumatic brain injury (TBI) patients [19], non-emergency department hospitalized patients [10], patients undergoing cardiac surgery [20], and patients with coronavirus disease (COVID-19) [21]. However, these prediction models were not focused on patients with sepsis so far. In this study, we developed a model based on several clinical indicators to predict the development of ARDS in sepsis patients admitted to ICU. The developed prediction model in this study contains thirteen predictors: BMI, respiratory rate, urine output, PCO2, BUN, vasopressin, CRRT, ventilation status, chronic pulmonary disease, malignant cancer, liver disease, septic shock and pancreatitis. Liver disease was regarded to be a predictor of developing ARDS in this study, which were consistent with prior studies [22, 23]. A study has expounded that liver disease was an important predictor for the in-hospital mortality of patients with sepsis and lung infection [22]. In general, the liver could prevent sepsis from aggravating tissue and organ damage by removing bacteria and regulating the metabolism of inflammatory factors. However, when the liver occurs injury, it might increase the inflammatory response of the lung to septic bacterial infection, which leading to an increased risk of ARDS [22, 23]. In the study of Li X, et al., respiratory rate in the non-survival group was significantly higher than that of the survival group among sepsis patients with developing ARDS, which also indicated that respiratory rate was associated with the prognosis for sepsis patients with developing ARDS [24].

Nowadays, nomogram has proven to be an effective tool in predicting an individual’s probability of a clinical event, and it is consistent with the requirements of integrated model [25]. Moreover, the nomogram is also simple, intuitive and convenient for clinicians to use on prognostic prediction of disease [26]. In the present study, for visualizing the developed prediction model, we plotted a nomogram. Additionally, the ROC curves indicated that this established model had a predictive ability compared with SOFA score and SAPS II score. It is worth noting that, we have also developed an online prediction system, which may be more convenient for clinical application (https://xuchi777.shinyapps.io/DynNomapp/). The developed predictive model may also be a potential tool to guide clinicians in predicting the risk of ARDS in septic patients in the ICU, which help take early interventions to prevent ARDS progression in sepsis patients admitted to ICU and improved clinical outcomes.

The present study had some strengths. Firstly, the relatively large sample size of this study makes the results convincing. Secondly, we developed a model with an intuitive and easy to use based on some clinical indicators to predict the ARDS risk of ICU patients with sepsis. Simultaneously, the result of internal validation showed that the prediction model had a good discrimination and accuracy in predicting the risk of ARDS for sepsis patients. Nevertheless, we also acknowledged that there were some limitations in this study. Firstly, due to all patients from MIMIC-IV database and only septic patients in ICU were considered, we were unable to confirm whether this developed prediction model was applicable to patients with sepsis who were not admitted to the ICU. More prospective studies are needed to validate this result. Secondly, this is a retrospective cohort study, some variables with more than 20% missing values (ALT, ALB, TG, LDL-C, HDL-C, AST, NEUT, SaO_2_, TC and CRP) were deleted, which may affect the result. Thirdly, MIMIC-IV is a single-center database, so the results of this study should be prudently interpreted when involving other populations. Lastly, an external validation should be still required in the future.

## Conclusion

In conclusion, we developed a prediction model incorporating thirteen clinical features to effectively predict the ARDS risk in ICU patients with sepsis. Additionally, the prediction model showed a good predictive ability as well as discrimination by internal validation. Nevertheless, further prospective studies are warranted to validate the effectiveness and applicability of this prediction model.

## Supplementary Information

**Additional file 1: Table S1.** The difference analysis between the training set and testing set

## Data Availability

The datasets generated and/or analyzed during the current study are available in the MIMIC-IV database, https://mimic.physionet.org/iv/.

## Abbreviations

ARDS: Acute respiratory distress syndrome
MIR103: MicroRNA 103
MIR107: MicroRNA 107
MIMIC: Medical Information Mart for Intensive Care
ICU: Intensive care unit
BIDMC: Beth Israel Deaconess Medical Center
BMI: Body mass index
SBP: Systolic blood pressure
DBP: Diastolic blood pressure
NEUT: Neutrophil
LYM: Lymphocyte
PLT: Platelet
WBC: White blood cell
ALB: Albumin
ALT: Alanine aminotransferase
AST: Aspartate aminotransferase
BUN: Blood urea nitrogen
CRP: C-reactive protein
TC: Total cholesterol
TG: Triglycerides
LDL-C: Low density lipoprotein cholesterol
HDL-C: High density lipoprotein cholesterol
SOFA: Sequential organ failure assessment
SAPS: Simplified acute physiology score
CRRT: Continuous renal replacement therapy
MV: Mechanical ventilation
CRRT: Continuous renal replacement therapy
PEEP: Positive end-expiratory pressure
ROC: Receiver operating characteristic
RR: Relative risk
CI: Confidence interval
