# Supplementary material for: A prediction model for predicting the risk of acute respiratory distress syndrome in sepsis patients: a retrospective cohort study
Source: BMC Pulm Med. 2023 Mar 8;23:78. doi: 10.1186/s12890-023-02365-z (PMC9994387; doi:10.1186/s12890-023-02365-z)
Supplement: Supplementary file 1 — Additional file 1: Table S1. The difference analysis between the training set and testing set [file 12890_2023_2365_MOESM1_ESM.doc]

**Supplemental Table 1. The difference analysis between the training set and testing set.**

| Variables | Total (n=16523) | Training set (n=11566) | Testing set (n=4957) | Statistics | *P* |
| --- | --- | --- | --- | --- | --- |
| Age, years, Mean ± SD | 65.48 ± 15.61 | 65.45 ± 15.61 | 65.56 ± 15.62 | t=-0.42 | 0.678 |
| Gender, n (%) |  |  |  | χ^2^=0.078 | 0.780 |
| Female | 6570 (39.76) | 4607 (39.83) | 1963 (39.60) |  |  |
| Male | 9953 (60.24) | 6959 (60.17) | 2994 (60.40) |  |  |
| BMI, kg/m^2^, n (%) |  |  |  | χ^2^=1.021 | 0.796 |
| < 18.5 | 482 (2.92) | 334 (2.89) | 148 (2.99) |  |  |
| ≥18.5 and <25 | 4740 (28.69) | 3322 (28.72) | 1418 (28.61) |  |  |
| ≥25 and <30 | 5389 (32.62) | 3749 (32.41) | 1640 (33.08) |  |  |
| ≥30 | 5912 (35.78) | 4161 (35.98) | 1751 (35.32) |  |  |
| Ethnicity, n (%) |  |  |  | χ^2^=1.215 | 0.545 |
| Black | 1286 (7.78) | 896 (7.75) | 390 (7.87) |  |  |
| Other/Unknown | 4081 (24.70) | 2831 (24.48) | 1250 (25.22) |  |  |
| White | 11156 (67.52) | 7839 (67.78) | 3317 (66.92) |  |  |
| Marital status, n (%) |  |  |  | χ^2^=2.943 | 0.230 |
| Married | 7738 (46.83) | 5448 (47.10) | 2290 (46.20) |  |  |
| Other/Unknown | 4675 (28.29) | 3227 (27.90) | 1448 (29.21) |  |  |
| Single | 4110 (24.87) | 2891 (25.00) | 1219 (24.59) |  |  |
| Insurance, n (%) |  |  |  | χ^2^=1.003 | 0.605 |
| Medicaid | 1133 (6.86) | 789 (6.82) | 344 (6.94) |  |  |
| Medicare | 7441 (45.03) | 5238 (45.29) | 2203 (44.44) |  |  |
| Other | 7949 (48.11) | 5539 (47.89) | 2410 (48.62) |  |  |
| Admission type, n (%) |  |  |  | χ^2^=2.736 | 0.434 |
| Elective | 1046 (6.33) | 739 (6.39) | 307 (6.19) |  |  |
| Emergency | 7978 (48.28) | 5588 (48.31) | 2390 (48.21) |  |  |
| Other | 3610 (21.85) | 2491 (21.54) | 1119 (22.57) |  |  |
| Urgent | 3889 (23.54) | 2748 (23.76) | 1141 (23.02) |  |  |
| SBP, mmHg, M (Q_1_, Q_3_) | 118.00 (104.00, 135.00) | 118.00 (104.00, 135.00) | 118.00 (105.00, 135.00) | Z=0.478 | 0.633 |
| DBP, mmHg, M (Q_1_, Q_3_) | 64.00 (54.00, 76.00) | 64.00 (54.00, 76.00) | 64.00 (54.00, 75.00) | Z=-0.585 | 0.559 |
| Temperature, ℃, Mean ± SD | 36.63 ± 0.94 | 36.63 ± 0.95 | 36.64 ± 0.92 | t=-0.96 | 0.337 |
| Heart rate, times/min, Mean ± SD | 89.30 ± 21.04 | 89.37 ± 20.02 | 89.12 ± 23.25 | t=0.66 | 0.512 |
| Respiratory rate, times/min, M (Q_1_, Q_3_) | 18.00 (15.00, 22.00) | 18.00 (15.00, 22.00) | 18.00 (15.00, 22.00) | Z=-1.222 | 0.222 |
| Urine output, mL, M (Q_1_, Q_3_) | 2980.00 (1890.00, 4405.00) | 2990.00 (1885.00, 4400.00) | 2960.00 (1905.00, 4430.00) | Z=-0.093 | 0.926 |
| PCO_2_, mmHg, Mean ± SD | 42.31 ± 11.93 | 42.33 ± 11.94 | 42.26 ± 11.89 | t=0.36 | 0.715 |
| FiO_2_, mmHg, M (Q_1_, Q_3_) | 100.00 (50.00, 100.00) | 100.00 (50.00, 100.00) | 100.00 (50.00, 100.00) | Z=0.440 | 0.660 |
| HCO_3_ -, Mean ± SD | 22.50 ± 4.66 | 22.52 ± 4.67 | 22.45 ± 4.63 | t=0.93 | 0.353 |
| Hemoglobin, g/dL, Mean ± SD | 11.38 ± 2.29 | 11.38 ± 2.28 | 11.38 ± 2.30 | t=0.01 | 0.994 |
| PLT, K/L, M (Q1, Q3) | 188.00 (135.00, 251.00) | 189.00 (135.00, 252.00) | 185.00 (134.00, 249.00) | Z=-1.283 | 0.199 |
| WBC, K/L, M (Q1, Q3) | 10.60 (7.40, 15.10) | 10.60 (7.40, 15.10) | 10.70 (7.50, 15.20) | Z=1.033 | 0.301 |
| Creatinine, mg/dL,M (Q_1_, Q_3_) | 1.00 (0.80, 1.50) | 1.00 (0.80, 1.50) | 1.00 (0.80, 1.50) | Z=0.801 | 0.423 |
| BUN, mg/dL, M (Q_1_, Q_3_) | 20.00 (14.00, 32.00) | 20.00 (14.00, 32.00) | 20.00 (14.00, 33.00) | Z=0.263 | 0.793 |
| Glucose, mg/dL, M (Q_1_, Q_3_) | 123.00 (101.00, 162.00) | 123.00 (101.00, 162.00) | 123.00 (101.00, 161.00) | Z=-0.113 | 0.910 |
| SOFA, M (Q_1_, Q_3_) | 39.00 (31.00, 49.00) | 39.00 (31.00, 49.00) | 38.00 (31.00, 49.00) | Z=-0.881 | 0.378 |
| SAPS II, M (Q_1_, Q_3_) | 2.00 (0.00, 4.00) | 2.00 (0.00, 4.00) | 2.00 (0.00, 4.00) | Z=-0.414 | 0.679 |
| Vasopressin, n (%) |  |  |  | χ^2^=0.582 | 0.445 |
| No | 14248 (86.23) | 9989 (86.37) | 4259 (85.92) |  |  |
| Yes | 2275 (13.77) | 1577 (13.63) | 698 (14.08) |  |  |
| CRRT, n (%) |  |  |  | χ^2^=0.799 | 0.371 |
| No | 15240 (92.24) | 10682 (92.36) | 4558 (91.95) |  |  |
| Yes | 1283 (7.76) | 884 (7.64) | 399 (8.05) |  |  |
| Ventilation status, n (%) |  |  |  | χ^2^=4.097 | 0.393 |
| High flow | 766 (4.64) | 557 (4.82) | 209 (4.22) |  |  |
| Invasive vent | 155 (0.94) | 114 (0.99) | 41 (0.83) |  |  |
| Non-Invasive vent | 3044 (18.42) | 2115 (18.29) | 929 (18.74) |  |  |
| Oxygen | 103 (0.62) | 73 (0.63) | 30 (0.61) |  |  |
| Trach | 12455 (75.38) | 8707 (75.28) | 3748 (75.61) |  |  |
| RBC-transfusion, n (%) |  |  |  | χ^2^=0.429 | 0.513 |
| No | 9820 (59.43) | 6855 (59.27) | 2965 (59.81) |  |  |
| Yes | 6703 (40.57) | 4711 (40.73) | 1992 (40.19) |  |  |
| PLT-transfusion, n (%) |  |  |  | χ^2^=0.069 | 0.793 |
| No | 14078 (85.20) | 9860 (85.25) | 4218 (85.09) |  |  |
| Yes | 2445 (14.80) | 1706 (14.75) | 739 (14.91) |  |  |
| Frozen plasma, n (%) |  |  |  | χ^2^=0.015 | 0.904 |
| No | 14381 (87.04) | 10069 (87.06) | 4312 (86.99) |  |  |
| Yes | 2142 (12.96) | 1497 (12.94) | 645 (13.01) |  |  |
| Diabetes, n (%) |  |  |  | χ^2^=2.660 | 0.103 |
| No | 11442 (69.25) | 7965 (68.87) | 3477 (70.14) |  |  |
| Yes | 5081 (30.75) | 3601 (31.13) | 1480 (29.86) |  |  |
| Chronic pulmonary disease, n (%) |  |  |  | χ^2^=0.246 | 0.620 |
| No | 12110 (73.29) | 8464 (73.18) | 3646 (73.55) |  |  |
| Yes | 4413 (26.71) | 3102 (26.82) | 1311 (26.45) |  |  |
| Renal disease, n (%) |  |  |  | χ^2^=0.047 | 0.828 |
| No | 13029 (78.85) | 9115 (78.81) | 3914 (78.96) |  |  |
| Yes | 3494 (21.15) | 2451 (21.19) | 1043 (21.04) |  |  |
| Malignant cancer, n (%) |  |  |  | χ^2^=0.564 | 0.453 |
| No | 14562 (88.13) | 10179 (88.01) | 4383 (88.42) |  |  |
| Yes | 1961 (11.87) | 1387 (11.99) | 574 (11.58) |  |  |
| Liver disease, n (%) |  |  |  | χ^2^=0.265 | 0.606 |
| No | 14057 (85.08) | 9829 (84.98) | 4228 (85.29) |  |  |
| Yes | 2466 (14.92) | 1737 (15.02) | 729 (14.71) |  |  |
| Myocardial infarct, n (%) |  |  |  | χ^2^=0.110 | 0.740 |
| No | 13249 (80.19) | 9282 (80.25) | 3967 (80.03) |  |  |
| Yes | 3274 (19.81) | 2284 (19.75) | 990 (19.97) |  |  |
| Leukemia, n (%) |  |  |  | χ^2^=2.157 | 0.142 |
| No | 16271 (98.47) | 11379 (98.38) | 4892 (98.69) |  |  |
| Yes | 252 (1.53) | 187 (1.62) | 65 (1.31) |  |  |
| Septic shock, n (%) |  |  |  | χ^2^=0.220 | 0.639 |
| No | 15612 (94.49) | 10922 (94.43) | 4690 (94.61) |  |  |
| Yes | 911 (5.51) | 644 (5.57) | 267 (5.39) |  |  |
| Pancreatitis, n (%) |  |  |  | χ^2^=0.048 | 0.827 |
| No | 16156 (97.78) | 11311 (97.80) | 4845 (97.74) |  |  |
| Yes | 367 (2.22) | 255 (2.20) | 112 (2.26) |  |  |
| ARDS, n (%) |  |  |  | χ^2^=1.906 | 0.167 |
| No | 13110 (79.34) | 9144 (79.06) | 3966 (80.01) |  |  |
| Yes | 3413 (20.66) | 2422 (20.94) | 991 (19.99) |  |  |

BMI, body mass index; SBP, systolic blood pressure; DBP, diastolic blood pressure; SPO_2_, pulse oxygen saturation; PCO_2_, partial pressure of carbon dioxide; PO_2_, oxygen partial pressure; FiO_2_, fraction of inspired oxygen; HCO_3_^-^, bicarbonate; PLT, platelet; WBC, white blood cell; BUN, blood urea nitrogen; OFA, sequential organ failure assessment; SAPS II, simplified acute physiology score II; CRRT, continuous renal replacement therapy; RBC, red blood cell; PLT, platelets; ARDS, acute respiratory distress syndrome.
